# Lymphoid interstitial pneumonia in Sjögren disease: clinical course and comparison with other ILD patterns

**DOI:** 10.1093/rheumatology/keag321

**Published:** 2026-06-22

**Authors:** Gaetano La Rocca, Francesco Ferro, Vincenzo Uggenti, Beatrice Dei, Giovanni Fulvio, Michele Moretti, Riccardo Morganti, Gianluca Sambataro, Marta Mosca, Chiara Romei, Chiara Baldini

**Affiliations:** Rheumatology Unit, Department of Clinical and Experimental Medicine, Azienda Ospedaliero Universitaria Pisana, University of Pisa, Pisa, Italy; Rheumatology Unit, Department of Clinical and Experimental Medicine, Azienda Ospedaliero Universitaria Pisana, University of Pisa, Pisa, Italy; Department of Translational Research, Academic Radiology, University of Pisa, Pisa, Italy; Rheumatology Unit, Department of Clinical and Experimental Medicine, Azienda Ospedaliero Universitaria Pisana, University of Pisa, Pisa, Italy; Rheumatology Unit, Department of Clinical and Experimental Medicine, Azienda Ospedaliero Universitaria Pisana, University of Pisa, Pisa, Italy; Rheumatology Unit, Department of Clinical and Experimental Medicine, Azienda Ospedaliero Universitaria Pisana, University of Pisa, Pisa, Italy; Section of Statistics, University of Pisa, Pisa, Italy; Department of Medicine and Surgery, University of Enna “Kore”, Enna, Italy; Rheumatology Unit, Department of Clinical and Experimental Medicine, Azienda Ospedaliero Universitaria Pisana, University of Pisa, Pisa, Italy; Department of Translational Research, Academic Radiology, University of Pisa, Pisa, Italy; Rheumatology Unit, Department of Clinical and Experimental Medicine, Azienda Ospedaliero Universitaria Pisana, University of Pisa, Pisa, Italy

**Keywords:** lymphoid interstitial pneumonia, Sjögren disease, interstitial lung disease, HRCT pattern, phenotypes

## Abstract

**Objectives:**

Lymphoid interstitial pneumonia (LIP) is a rare form of Interstitial Lung Disease (ILD), often associated with Sjögren Disease (SjD). However, the clinical-serologic characteristics of SjD-LIP remain poorly characterized. Our objective was to describe the clinical course and outcome of SjD-associated LIP and to compare this subgroup with other SjD-ILD patterns.

**Methods:**

SjD patients with High-resolution Computed Tomography (HRCT)-confirmed ILD followed in Pisa Rheumatology Unit (January 2019–November 2024) were retrospectively enrolled. ILD patterns were classified through multidisciplinary discussion. Clinical and laboratory data were collected according to ESSDAI definitions, along with pulmonary symptoms and function tests (PFTs).

**Results:**

Fifty-five SjD-ILD patients were included (M: F = 9:46), of whom 11 were diagnosed with LIP (F: M = 11:0). LIP patients showed thin-walled parenchymal cysts as the predominant HRCT finding, and largely preserved pulmonary function (median forced vital capacity [FVC] 101% [IQR 98–105]; DLCO 76% [IQR 75–81]). After a median 5-years follow-up (IQR 2–7) all LIP patients were alive with stable PFTs. Compared with the remaining 44 non-LIP, LIP patients were younger at SjD diagnosis (*P* < 0.001) and more frequently presented purpura (*P* = 0.012), constitutional symptoms (*P* = 0.001), lymphadenopathy (*P* = 0.023), hypergammaglobulinemia (*P* < 0.001), triple anti-Ro60/52/La positivity (*P* = 0.035) and C3 hypocomplementemia (*P* = 0.009). ILD preceded SjD diagnosis in 30/44 non-LIP vs. 1/11 LIP patients (*P* < 0.001), with lower FVC% (*P* = 0.049) and DLCO% (*P* = 0.036) in non-LIP.

**Conclusion:**

LIP defines a distinct, immunologically active phenotype within the spectrum of SjD-ILD, characterized by greater extrapulmonary systemic involvement and serologic markers of B cell hyperactivity, but limited pulmonary functional impact. These findings support long-term lymphoma surveillance and a potential role for B cell targeted therapies in selected patients.

Rheumatology key messages

- LIP in SjD identifies a distinct ILD phenotype with prominent extrapulmonary systemic involvement and immunologic activity.
- Increase in cyst burden in LIP does not necessarily translate into worse pulmonary outcomes.
- B-cell hyperactivity in LIP may represent both a therapeutic target and a rationale for long-term lymphoma surveillance.

## Introduction

Sjögren Disease (SjD) is an autoimmune systemic disorder belonging to the family of CTDs and characterized by lymphocytic hyperactivation and proliferation [1]. Auto-reactive lymphocytes target epithelial cells in salivary and lacrimal glands, establishing a complex interplay which leads to glandular hypofunction and ultimately to irreversible fibrotic damage [2, 3].

SjD is also known as a ‘systemic epithelitis’ since the same pathophysiologic events potentially affect different systems and organs presenting as extra-glandular manifestations in up to 40% of patients [4]. Particularly, lung involvement is increasingly recognized as a common manifestation of the disease, significantly increasing the morbidity and mortality burden of affected patients, as well as quality of life [5]. Compared with other CTDs, Sjögren’s disease-related interstitial lung disease (ILD) is particularly heterogeneous in terms of imaging findings, clinical course and prognosis [6], due to the potential coexistence of small airways disease and lymphoproliferative lesions [7].

Based on their clinical behaviour, radiologic and histologic findings, CTD-related ILDs are usually categorized in different patterns resembling their idiopathic counterparts according to the widely accepted classification of Idiopathic Interstitial Pneumonias from the ATS/ERS [8]. Of note, nowadays High-resolution Computed Tomography (HRCT) is regarded as the gold standard technique for diagnosing and characterizing CTD-ILD and lung biopsy is seldom required for diagnostic purposes in the proper clinical scenario [9].

First described by Lebow and Carrington in 1966, Lymphoid Interstitial Pneumonia (LIP) is a very rare condition, classified both among idiopathic ILDs and benign pulmonary lymphoproliferative disorders [10]. It is characterized histologically by a diffuse lymphocytic infiltrate expanding alveolar and interlobular septa, often accompanied by airway centred lymphocytic inflammation and BALT hyperplasia [11]. SjD is among the most common causes of LIP, followed by immunodeficiency syndromes (including AIDS and CVID), chronic viral infections and other systemic and organ-specific autoimmune diseases. On the other hand, LIP is regarded to account for up to 10–20% of SjD-ILD cases [12].

Despite this association, due to the rarity of LIP, most of the available data on this condition is derived by pathology and/or radiology series of LIP patients with various underling aetiologies [13–15]. In these studies, information on the diagnosis and clinical features of SjD patients is limited, with the notable exception of a recent study conducted in China [16].

As a result, the clinical, immunologic, and prognostic profile of SjD-associated LIP remains poorly characterized.

In this study, we aimed to systematically characterize the clinical, serologic, and radiologic features of SjD-LIP and to compare them with other ILD patterns in a well-defined single-centre cohort. Our goal was to explore whether LIP defines a distinct phenotype of pulmonary involvement in SjD with specific implications for disease monitoring and prognosis.

## Methods

### Patients’ enrolment

This was a single-centre retrospective cohort study, including consecutive SjD patients with HRCT-proven ILD in follow-up from January 2019 to November 2024 in the Rheumatology department of the University Hospital of Pisa (AOUP), Italy.

All patients included in the study fulfilled the ACR/EULAR 2016 criteria for the classification of SjD [17] and cases of SjD overlapping with another CTD were systematically excluded. Only patients with complete autoantibody testing by EUROLINE immunoblot assay (EUROIMMUN, Germany) for CTD were included.

The study was conducted in accordance with the Declaration of Helsinki, and approved by the Ethical Committee ‘Comitato Etico Regione Toscana—Area Vasta Nord Ovest (CEAVNO)’ (protocol code: AIS-ILD; date of approval: 23 May 2024). Written informed consent was obtained from all participants.

### ILD definition and characterization

ILD diagnosis was based on clinical, functional, and radiological findings, and confirmed by chest HRCT scans review by two expert thoracic radiologists. In patients with fibrotic abnormalities on HRCT, the extent of interstitial involvement was visually estimated using a semi-quantitative scoring approach based on predefined anatomical levels, with the percentage of fibrotic involvement assessed across representative thoracic sections and averaged to obtain the overall extent of disease, according to previously described methods [18]. ILD patterns were classified as usual interstitial pneumonia (UIP), non-specific interstitial pneumonia (NSIP), organizing pneumonia (OP), lymphocytic interstitial pneumonia (LIP) or unclassifiable (NC) according to the American Thoracic Society/European Respiratory Society classification [8]. A radiological LIP pattern was defined based on combinations of the pertinent radiologic findings—namely ground glass opacities, scattered thin-walled cysts, reticular changes and centrilobular nodules—when alternative diagnoses (particularly NSIP) were considered less likely and excluded through multidisciplinary discussion.

Histological data were available for two patients who underwent open lung biopsy to rule out lymphoma due to the presence of large pulmonary nodules. In both cases, histopathology and immunohistochemistry findings were consistent with pulmonary amyloidosis superimposed on LIP.

For patients with available HRCT scans of suitable technical characteristics, an exploratory computer-aided evaluation with Computer-Aided Lung Informatics for Pathology Evaluation and Rating (CALIPER) was performed, as previously described [19].

### Data collection and study design

For all patients, demographic data, clinical and laboratory manifestations at diagnosis and during follow-up were collected based on the EULAR Sjögren’s syndrome disease activity index (ESSDAI) [20]. Regarding glandular involvement, the presence of xerostomia and xeropthalmia, graded on a 0–10 Visual Analogue Scale (VAS), Schirmer’s test results and major Salivary Glands Ultra Sound (SGUS) findings graded according to the Omeract 0–3 grey scale score were recorded. Minor salivary gland biopsy results, including focus score where available, were also collected. Presence of extra-glandular organ involvement was defined according to ESSDAI definitions [20].

Regarding the respiratory picture, presence of chronic cough (defined as persistent cough for >8 weeks, according to the European Respiratory Society [21, 22]) and dyspnoea (defined as present with a score ≥2 according to the modified Medical Research Council dyspnoea scale [23]), smoke exposure at the time of ILD diagnosis, and the time interval between SjD and ILD diagnosis were recorded. Pulmonary Function Tests (PFT) results including forced vital capacity (FVC) and diffusing capacity of the lung for carbon monoxide (DLCO) defined as % of predicted values at the time of ILD diagnosis and at the last available follow-up were collected.

For LIP patients, total number of cysts and the maximum diameter of the largest cyst on HRCT were recorded at baseline and at the last available radiologic imaging to assess damage progression.

Finally, the clinical, serologic, functional and imaging characteristics were compared between LIP and non-LIP primary SjD-ILD patients.

### Statistical analysis

Data were expressed as mean (±SD) or median (IQR) for normally distributed and not normally distributed continuous variables, respectively, and as absolute frequencies and percentages for categorical variables. To analyse categorical and continuous variables *χ*^2^ test or exact Fisher test and Student’s *t* test or Mann–Whitney test were used, respectively. Furthermore, Wilcoxon test was applied to compare paired data. Significance was set at 0.05 and all the analyses were performed by SPSS v.29 technology.

## Results

### Study population

Out of 496 primary SjD patients in follow-up from January 2019 to November 2024, 55 patients (M: F = 9:46) with HRCT-confirmed ILD were retrospectively enrolled. All the included patients fulfilled the 2016 ACR/EULAR classification criteria. The mean age at the time of SjD diagnosis was 59.2 (±15.5) years, while the mean disease duration at the time of the study was 10.7 (±10.9) years.

Antinuclear autoantibodies (ANA) assessed by indirect immunofluorescence were positive in 54 (98.2%) cases; Regarding specific autoantibodies: 21 (38.2%) patients tested positive for anti-Ro60/52 and anti-La/SSB antibodies; 13 (23.6%) were anti-Ro60/52 positive, 12 (21.8%) presented isolated anti-Ro52 positivity, 1 isolated anti-Ro60 (1.8%) and 8 (14.5%) were seronegative for anti-Ro/La but met SjD criteria based on ocular tests and positive minor salivary gland biopsy.

SjD preceded ILD diagnosis in 31 (56.4%) patients, with a mean latency between SjD and ILD diagnosis of 5.8 (±10.8) years. For the remaining 24 (43.6%) cases, ILD diagnosis anticipated—or occurred concomitantly with—SjD diagnosis.

ILD pattern on HRCT was defined as NSIP in 23 (41.8%), LIP in 11 (20%), NSIP+OP in 9 (16.4%), UIP in 9 (16.4%) and OP in 2 (3.6%) patients.

### LIP patients

Eleven out of 55 SjD-ILD patients presented radiologic findings on HRCT consistent with a radiologic LIP pattern (F: M = 11:0) and were classified as LIP following exclusion of alternative aetiologies (particularly infectious) and multidisciplinary discussion.

The median age at SjD diagnosis of LIP patients was 37 (IQR 27–51) years and in all but one cases ILD diagnosis followed SjD diagnosis with a median latency of 11 (IQR 6–36) years.

Sicca symptoms of moderate intensity were present in all patients at SjD diagnosis, being the mean VAS 6/10 (±2.1) and 5.6/10 (±2.0) for xeropthalmia and xerostomia, respectively. Overall, LIP patients presented a moderate symptom burden, with a mean ESSPRI of 5.67 (±2.4).

Extra-glandular manifestations beyond pulmonary involvement occurred in 10 out of 11 patients. The most frequently involved ESSDAI domains included the articular (63.6%), hematologic (54.5%), lymphnodal (45.5%) and cutaneous domains (36.4%).

Regarding laboratory data, all LIP patients tested positive for ANA and anti-SSA, namely 7 patients with triple positivity for anti-Ro60/52/La (64%) and 4 patients with positivity of anti-Ro60/52 (36%); RF was positive in 7 (64%) and all patients presented hypergammaglobulinemia (100%), with 3 patients presenting also a monoclonal component (27%). Low serum levels of C3 and C4 fractions were detectable in 5 (45.5%) and 4 (36.4%) patients, respectively.

Minor salivary gland biopsy was available in 7/11 LIP patients, showing a median focus score of 3.2 (IQR 1.6–3.6), consistent with marked lymphocytic glandular infiltration.

Complete demographic, clinical, and serologic characteristics of LIP patients are shown in Supplementary Table S1.

### Clinical and radiologic features of LIP

At the time of LIP diagnosis, 10 out of 11 (91%) patients had a history of recurrent bronchopneumonia episodes presenting with acute dyspnoea and cough, often accompanied by fever and typically resolving with systemic corticosteroid therapy. Six of these patients required at least one hospitalization. In contrast, chronic exertional dyspnoea and chronic cough were much less common at the time of LIP diagnosis, being present in 3 (27.3%) and 5 (45.5%) patients, respectively.

In one 70-year-old female patient, LIP was the first manifestation of SjD, presenting with ARDS requiring admission to ICU and treatment with high-dose pulse steroids.

Complete PFT results at ILD diagnosis and last follow-up were available for 9 patients. Of note, median FVC at baseline was 101% of predicted (IQR 93–110) with only one patient presenting a mild reduction of lung volumes (60<FVC < 80%). Median DLCO was 79% of predicted (IQR 75–84.5), with 6 patients presenting a mild impairment of pulmonary gas transfers (60<DLCO < 80%). Finally, 3 patients exhibited a mild obstructive defect (FEV1/FVC < 80) on spirometry.

Regarding HRCT findings, a total of 39 scans were available and analysed for all of the 11 LIP patients enrolled. Scattered, thin-walled parenchymal cysts were the most common radiological feature, being present in all cases (100%). The median number of cysts on baseline HRCT was 23.5 (IQR 9.5–57), with the largest cysts presenting a median maximum diameter of 21 mm (IQR 14–50). All but one patient also presented signs of small airways involvement due to centrilobular and/or branching opacities with or without mosaic attenuation (90.9%). Five patients (45.5%) presented diffuse bilateral ground glass opacities and intra- and inter-lobular septal thickening, while pulmonary nodules were present in 4 cases (36.4%). Among patients with quantifiable fibrotic abnormalities on HRCT, the visually estimated extent of interstitial involvement was limited, with a median baseline extent of 4.5% and no relevant change during follow-up.

Histologic data were available only for 2 patients who underwent open lung biopsy due to the presence of large nodules raising the suspect of lymphoproliferative lesions. Histologic findings revealed the presence of nodular AL amyloidosis superimposed on LIP in both cases.

Table 1 summarizes the clinical, radiological and functional characteristics of ILD in LIP patients, as well as the treatment regimens administered.

**Table 1:** Clinical presentation, baseline HRCT pattern, pulmonary function and treatment of SjD-associated LIP patients.

Pt	Respiratory symptoms	Baseline HRCT findings					Baseline PFTs		Treatment
		Cyst	SA	GGO	Septa	Nodules	FVC %predicted	DLco %predicted	
1	Chronic dyspnoea, recurrent BP-E	+	+	–	–	+	100	72	GC
2	Chronic cough, recurrent BP-E	+	+	–	–	–	115	90	GC, MMF
3	Recurrent BP-E	+	+	–	–	–	164	88	GC
4	Chronic cough, recurrent BP-E	+	–	+	+	–	88	76	GC, RTX
5	Recurrent BP-E	+	+	+	+	–	77	81	GC, RTX
6	Recurrent BP-E	+	+	+	+	–	107	75	GC
7	Chronic dyspnoea	+	+	–	–	+	105	79	GC, RTX
8	Recurrent BP-E	+	+	+	+	+	101	76	GC, BEL
9	Chronic cough, recurrent BP-E	+	+	–	–	–	103	74	AZA
10	Chronic cough, recurrent BP-E	+	+	–	–	+	NA	NA	GC
11	Chronic dyspnoea, chronic cough, recurrent BP-E	+	+	+	+	–	NA	NA	GC, RTX

Respiratory symptoms shown represent the clinical presentation at the time of LIP diagnosis and the clinical indications that led to HRCT performance in patients with established SjD. In one case, acute respiratory failure prompted chest CT and led to the diagnosis of both LIP and SjD. BP-E: BronchoPneumonia- Episodes; SA: Small Airway involvement signs; GGO: Ground-Glass Opacities; GC: Glucocorticoids; MMF: Mycophenolate Mofetil; RTX: Rituximab; BEL: Belimumab; AZA: Azathioprine.

### LIP clinical course and outcome

Following LIP diagnosis, 10 (91%) patients were treated with systemic glucocorticoids and 7 (63.6%) received steroid-sparing immunosuppressive treatment, namely rituximab in 4 cases, azathioprine, mycophenolate mofetil and belimumab in 1 case each. Glucocorticoids were mainly prescribed as induction therapy, followed by tapering according to clinical response. In most patients, steroids were discontinued within 6–12 months, while three patients received low-dose maintenance for ∼3 years; one patient with acute respiratory failure received intravenous methylprednisolone pulses.

After a median follow-up of 5 (IQRR 2–7) years all patients were alive and none required lung transplantation nor long-term Oxygen therapy (LTOT). Additionally, no patients developed pulmonary hypertension. At the last follow-up pulmonary symptoms were stable or improved in 8/11 patients, 2 patients developed new-onset chronic dyspnoea and 1 presented new-onset chronic cough during follow-up.

Regarding functional tests, at the last follow-up LIP patients exhibited a median FVC of 101% of predicted (IQR 86.5–114) and a median DLCO of 79% of predicted (IQR 76–81).

**Figure 1:**
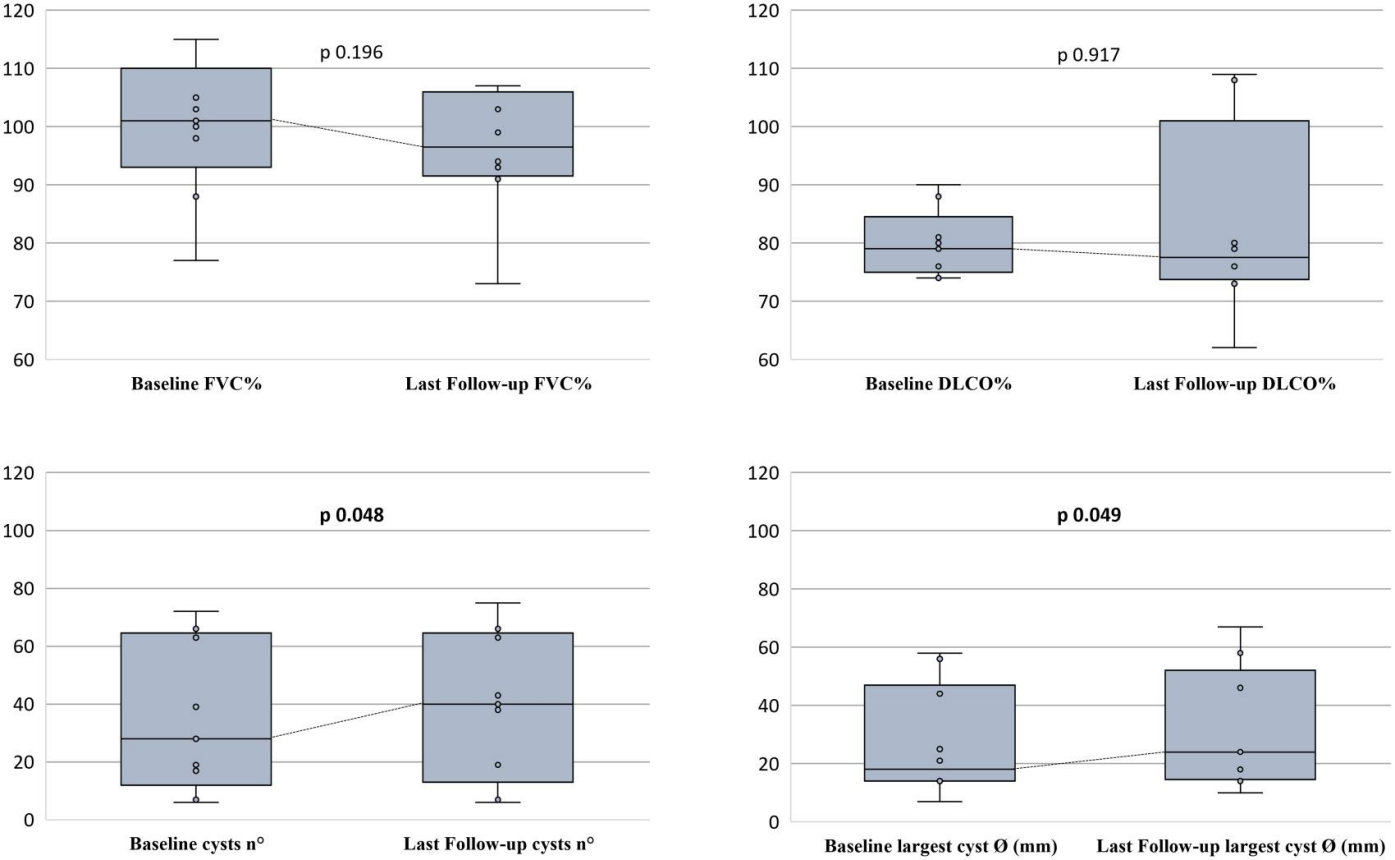
Box plots illustrating the median (IQR) of PFTs values, total cysts number and maximum diameter on HRCT of LIP patients at baseline and last follow-up. Statistical differences between the two time points were assessed with the Wilcoxon signed-rank test. Upper left: median FVC%predicted was 101 (93–110) at baseline and 96.5 (92–105) at last follow-up, respectively (*P* = 0.196). Upper right: median DLCO%predicted was 79 (75–84.5) at baseline and 77.5 (73.75–101) at last follow-up, respectively (*P* = 0.917). Lower left: median total number of parenchymal cysts was 23.5 (7–63) at baseline and 40 (13–64.5) at last follow-up, respectively (*P* = 0.048). Lower right: median of the maximum diameter of the largest cyst was 15 (14–50) at baseline and 24 (14.5–52) at last follow-up, respectively (*P* = 0.049)

Follow-up HRCT were available for 10/11 patients and the median time between baseline HRCT and the last HRCT available for each patient was 3 years (IQR 1–4). Of note in 4 patients (40%), both the total number of cysts and the maximum diameter of the largest cysts increased, in 1 patient (10%) the total number of cysts remained stable but the largest cyst was enlarged. The remaining 5 patients (50%) showed no radiologic progression. Overall, both the total number of cysts (*P* = 0.047) and the maximum diameter of the largest cysts (*P* = 0.048) were significantly increased between the baseline and the last HRCT (Fig. 1).

When compared with radiologically stable patients -and despite a similar pharmacological treatment and HRCT follow-up time-, those with an increase of cyst number and/or size did not show significant differences in terms of worsening of pulmonary symptoms and PFTs.

**Figure 2:**
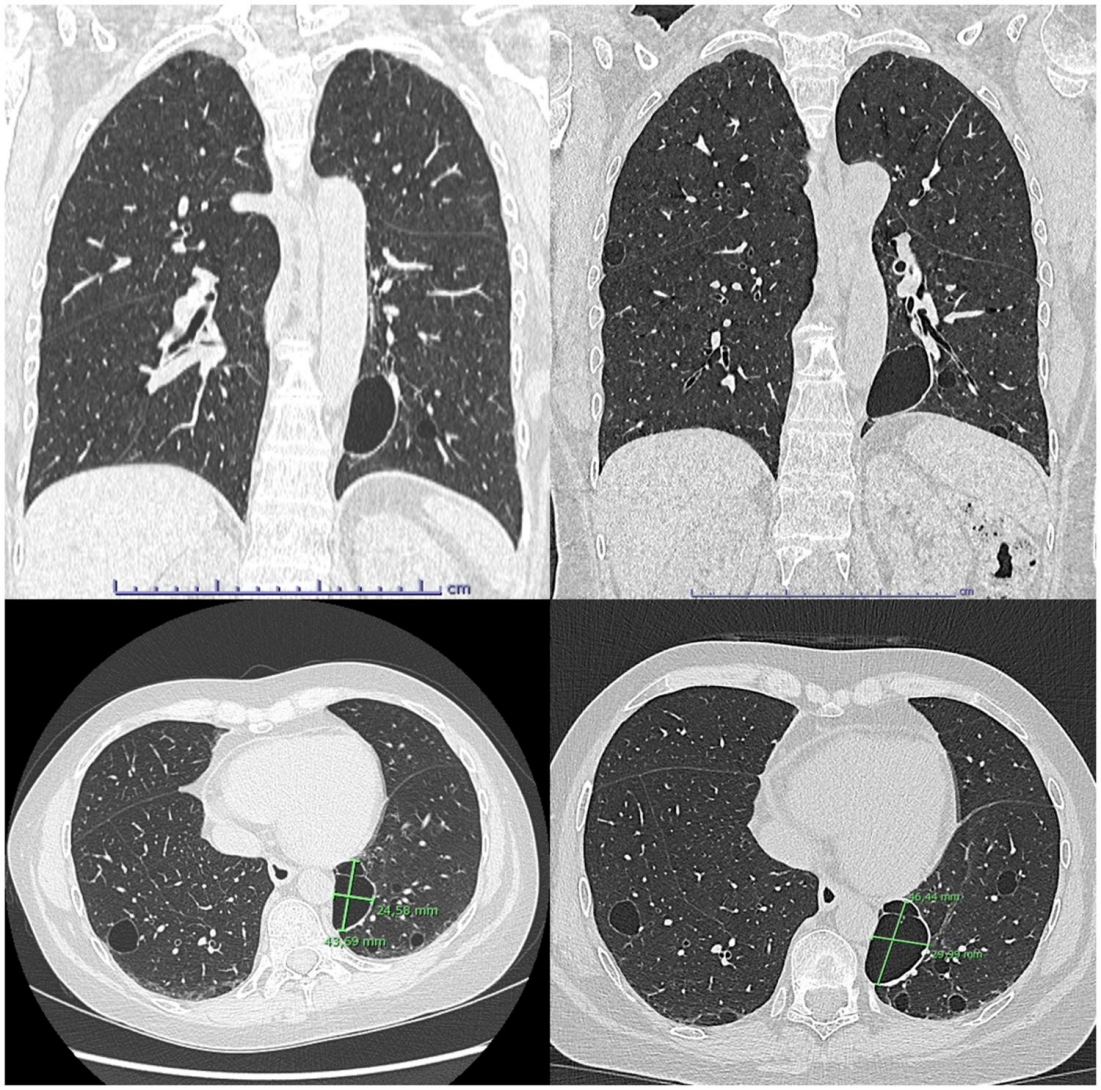
Images from a 50-year-old female patient with SjD-LIP. Top row: baseline and follow-up (after 32 months) coronal CT sections showing an increased number of cysts. Bottom row: measurements of the largest cyst, with diameters of 44 × 25 mm at baseline and 46 × 30 mm at follow-up

**Figure 3:**
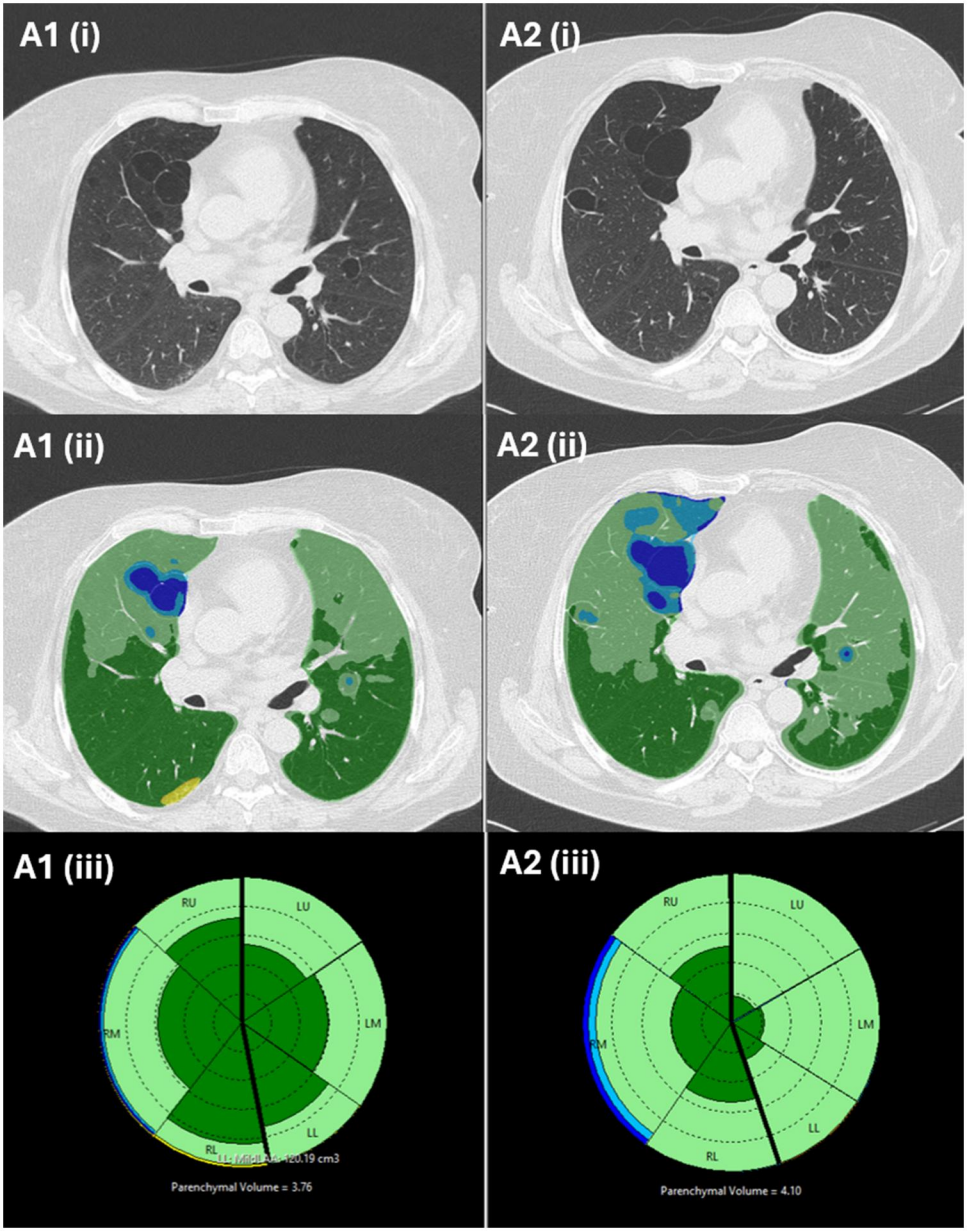
Example of CALIPER analysis of a patient with SjD-ILD. Axial HRCT slices (i), axial CALIPER-derived colour image overlays (ii) and Glyph of the lung parenchymal patterns (iii) as various colors [dark green = normal lung, light green = mild low attenuation areas (LAA), light blue: moderate LAA, blue = severe LAA] are shown. Interestingly, severe LAA accurately identify cystic areas providing an exact and reproducible measure of their extent compared with total lung parenchyma. A1 shows CALIPER evaluation at baseline with a severe LAA of 0.32%. A2 shows CALIPER evaluation at 30 months-follow-up with a severe LAA of 1.04%. The delta in the ‘severe LAA’ value, representing cystic disease progression in our case, was 0.72%

Examples of the visual and CALIPER-assisted evaluation of cysts progression on HRCT are shown in Figs 2 and 3, respectively.

### Comparison of SjD patients with LIP and other ILD patterns

Compared with non-LIP SjD patients, LIP patients were younger at the time of SjD and ILD diagnosis (*P* < 0.001 and 0.003, respectively).

Regarding glandular involvement, we observed no significant differences between the two groups in terms of subjective sicca symptoms (rated on a 0–10 VAS scale), objective ocular tests and SGUS OMERACT score in grey scale. Although a higher percentage of LIP patients presented with salivary gland enlargement, the difference did not reach statistical significance (*P* = 0.089). Similarly, there were no significant differences for the other cardinal subjective symptoms (fatigue and pain rated on a 0–10 VAS scale) and the overall ESSPRI score (Supplementary Table S2).

When analysing pulmonary involvement, ILD diagnosis more often anticipated SjD diagnosis in non-LIP patients (30/44 vs. 1/11). Among patients who developed ILD after SjD diagnosis, the median latency between SjD and ILD diagnosis was significantly longer in LIP patients (*P* = 0.003), despite comparable prior exposure to systemic glucocorticoids and DMARDs between LIP and non-LIP groups.

Chronic respiratory symptoms were significantly more prevalent in non-LIP patients. Chronic dyspnoea was reported in 34/44 (77.3%) non-LIP patients compared with 3/11 (27.3%) LIP patients (*P* = 0.003). Chronic cough was also more frequent in the non-LIP group (78.6%, vs. 45.5%), although this difference did not reach statistical significance (*P* = 0.056).

Moreover, non-LIP patients presented significantly lower values of FVC%predicted (*P* = 0.049) and DLCO%predicted (*P* = 0.036) on PFT performed at ILD diagnosis.

Regarding imaging findings, the visually estimated extent of fibrotic abnormalities on HRCT was low in LIP patients (median 4.5%), whereas a median value of 12.5% was observed in non-LIP patients.

Following ILD diagnosis, treatment strategies were comparable between the two groups in terms of glucocorticoid and immunosuppressive use (*P* = ns for both comparisons; Supplementary Table S3).

Regarding non-pulmonary extraglandular manifestations, LIP patients exhibited more often constitutional symptoms (*P* = 0.001), lymphnodal involvement (*P* = 0.023) and purpura (*P* = 0.012).

Finally, LIP patients showed a higher prevalence of hypergammaglobulinemia (*P* < 0.001), triple anti-Ro60/52/La positivity (*P* = 0.035) and low C3 complement levels (*P* = 0.009). RF positivity was also more common in LIP patients, although this did not reach statistical significance (*P* = 0.052).

Clinical and serologic comparisons between LIP and non-LIP patients are detailed in Tables 2 and 3, respectively.

**Table 2:** Comparison of clinical features of LIP and other ILD patterns.

Clinical features	LIP (*n* = 11)	Other ILD patterns (*n* = 44)	*P*-value
Age at SjD diagnosis, *m* (IQR)	37 (27–51)	66 (57–72)	**<0.001**
Age at ILD diagnosis, *m* (IQR)	58 (41–67)	69 (64–74)	**0.003**
SjD-ILD diagnosis latency, *m* (IQR)	11 (6–36)	0 (−0.5 to 6)	**0.003**
Sex male, *n* (%)	1 (9.1)	8 (18.2)	0.699
Smoking history, *n* (%)	0 0	8 (18.2)	0.188
FVC %, *m* (IQR)	101 (98–105)	86.5 (76–101)	**0.049**
DLCO%, *m* (IQR)	79 (76–81)	64 (49–79)	**0.036**
Constitutional symptoms, *n* (%)	4 (36.4)	0 0	**0.001**
Lymphnodal involvement, *n* (%)	6 (54.5)	9 (20.4)	**0.023**
- Non-Hodgkin Lymphoma, *n* (%)	0 0	2 (4.5)	1
Salivary gland enlargement, *n* (%)	6 (54.5)	9 (20.5)	0.089
Articular involvement, *n* (%)	2 (18.2)	4 (9.1)	0.588
Cutaneous involvement, *n* (%)	3 (27.3)	4 (9.1)	0.134
- Purpura, *n* (%)	4 (36.4)	2 (4.5)	**0.012**
Renal involvement, *n* (%)	1 (9.1)	3 (6.8)	1
Muscular involvement, *n* (%)	1 (9.1)	0 0	0.2
PNS involvement, *n* (%)	0 0	2 (4.5)	1
CNS involvement, *n* (%)	0 0	1 (2.3)	1
Hematologic involvement, *n* (%)	5 (45.5)	9 (20.4)	0.124
Biological activity, *n* (%)	11 (100)	23 (52.3)	**0.004**
Raynaud’s phenomenon, *n* (%)	5 (45.5)	20 (45.4)	1

SGE: salivary glands enlargement; PNS: peripheral nervous system; CNS: central nervous system; Presence of SGE and extra-glandular pSS manifestations was defined according to ESSDAI definitions.

Statistically significant *P* value are reported in bold.

**Table 3:** Comparison of laboratory features of LIP and other ILD patterns.

Laboratory findings	LIP (*n* = 11)	Other ILD patterns (*n* = 44)	*P*-value
Lymphopenia, *n* (%)	5 (45)	3 (7)	**0.004**
Hypergammaglobulinemia, *n* (%)	11 (100)	18 (41)	**<0.001**
MC, *n* (%)	3 (27.3)	6 (13.6)	0.362
C3 hypocomplementemia, *n* (%)	5 (45.5)	5 (11.4)	**0.009**
C4 hypocomplementemia, *n* (%)	4 (36.4)	6 (13.6)	0.099
Cryoglobulinemia, *n* (%)	1 (9.1)	3 (6.8)	1
Double anti-Ro60/52, *n* (%)	11 (100)	21 (47.7)	**0.002**
Isolated anti-Ro52, *n* (%)	0 0	8 (18.2)	0.188
Isolated anti-Ro60, *n* (%)	0 0	1 (2.3)	0.614
Anti-Ro seronegative, *n* (%)	0 0	8 (18.2)	0.126
Anti-La, *n* (%)	7 (63.6)	13 (29.5)	**0.035**
RF, *n* (%)	7 (63.6)	14 (31.8)	0.052

MC: monoclonal component; RF: rheumatoid factor.

Statistically significant *P* value are reported in bold.

## Discussion

To the best of our knowledge, this is the first study to describe in detail the clinical, immunologic, and radiologic features of LIP in a well-characterized Caucasian cohort of patients with SjD, with a median follow-up of five years. Importantly, we also compared this subgroup with other ILD patterns in SjD, highlighting distinctive phenotypic, serologic, and prognostic features.

Of note, SjD-LIP patients in our cohort exhibited a relatively homogeneous and distinctive phenotype. First, they were significantly younger at the time of SjD diagnosis with a median age of 37 years, which is quite close to the 35 years cut-off previously proposed to define Juvenile-onset SjD [24]. This finding contrasts with the well-established association between ILD and older age in SjD [25–27], which was also confirmed in our non-LIP subgroup.

Moreover, in ten out of eleven LIP cases, ILD diagnosis occurred after the diagnosis of SjD. In contrast, when considering ILD patients as a whole group, ILD preceded SjD diagnosis in almost half of the cases, in line with the available literature [28]. This observation suggests that LIP may arise as part of a more evolved and systemically active disease stage, rather than as an early disease manifestation.

Regarding the pulmonary picture, LIP presented more commonly with recurrent bronchopneumonia episodes without an identifiable infectious aetiology and highly responsive to systemic corticosteroids, probably reflecting acute lymphocytic inflammation of the bronchoalveolar complex. In contrast, chronic dyspnoea and cough, which are the typical symptoms of SjD-ILD, were much less common in LIP patients at diagnosis and only seldom developed during the disease course. Of note, this pattern of clinical behaviour may at least partly explain the longer latency between SjD and lung involvement diagnosis in LIP patients compared with non-LIP ILD cases.

HRCT findings were consistent with the radiologic definition of LIP, with bilateral thin-walled cysts with scattered distribution being the most common abnormality, similarly to the subgroup of SjD patients described in previous radiologic LIP series [12, 29].

Moreover, lung volumes on spirometry were mostly preserved in LIP patients, with the most common functional defect at diagnosis being a mild reduction of the diffusion capacity. In contrast, non-LIP patients exhibited significantly lower FVC and DLCO values, suggesting greater functional impairment. These findings are in line with those of Dong *et al.* on a Chinese cohort of SjD-LIP patients [16].

Longitudinal follow-up over a median of 5 years demonstrated an overall favourable pulmonary outcome in LIP patients. Most of them presented either improvement or stability of pulmonary symptoms and PFT parameters following treatment with glucocorticoids and immunosuppressants. Despite this, radiologic progression was observed, with significant increases in both cyst number and size. However, this did not correlate with worsening of pulmonary function or symptoms, suggesting that structural changes may reflect slow damage accrual with limited clinical impact.

The limited extent of fibrotic abnormalities observed in our cohort should be interpreted in light of recent radiologic-pathologic studies showing that so-called ‘radiologic LIP’, particularly in SjD, often reflects a heterogeneous spectrum of lymphoid proliferations frequently characterized by airway-centred inflammation rather than diffuse interstitial involvement [30].

We also evaluated the severity of cardinal subjective symptoms of SjD, namely sicca, fatigue and pain, showing no significant differences in these domains as well as in the overall symptom burden measured with ESSPRI between LIP and other ILD patients. Similarly, we observed no significant differences in glandular involvement with the exception of a tendency for more frequent SGE in LIP patients. In contrast, in this study we highlighted for the first time a higher frequency of extrapulmonary systemic manifestations in LIP patients compared with other ILD patients, including constitutional symptoms, purpura and lymphadenopathy. These findings may reflect a higher immunologic activity in LIP patients. Further supporting this hypothesis, all LIP patients were seropositive for anti-Ro60/52, with triple positivity for anti-Ro60/52/La detected in 7 out of 11 patients. Moreover, we observed a significantly higher prevalence of hypergammaglobulinemia, lymphopenia and low serum C3 levels in SjD-LIP, as well as a trend for RF positivity.

Interestingly, some of the distinguishing clinical and serologic features of LIP patients identified in our study are previously recognized risk factors for Non-Hodgkin lymphoma (NHL) in SjD [31]. However, none of LIP patients in our cohort developed clinically evident NHL. Nevertheless, considering the very slow kinetics leading from benign lymphoproliferation to the selection of malignant clones and clinical evidence of NHL in SjD [32], we cannot exclude that a longer follow-up would lead to the identification of subclinical lymphoproliferative evolution in LIP patients, particularly arising in the bronchus-associated lymphoid tissue (BALT-lymphoma).

Our study has some limitations. First of all, due to the retrospective nature of the study, caution is needed in the generalization of our findings. In addition, given the limited sample size, particularly in the LIP subgroup, all comparative analyses should be interpreted with caution and considered exploratory. On the other side, the relatively small number of LIP patients enrolled may have limited statistical power, preventing the identification of additional differences with non-LIP patients. Moreover, histologic confirmation of LIP diagnosis was available only for two patients who underwent open lung biopsy to exclude pulmonary lymphoma. However, the study also has different points of strength. Despite its retrospective design, it was conducted on a well characterized SjD cohort, which helped to limit the amount of missing data. Moreover, while histologic confirmation of LIP was not available for most patients, the pattern was defined based on multiple HRCT scans for each patient by two thoracic radiologists with expertise in ILD and following multidisciplinary discussion to exclude alternative aetiologies (particularly infectious causes). Importantly, there is some evidence that LIP secondary to autoimmune diseases defined by HRCT may present histologic differences compared with the pathologically defined LIP, reflecting a broader and potentially heterogeneous spectrum of lymphoid lung involvement in this setting [30]. However, HRCT is currently the gold-standard for the assessment of ILD in CTD patients, therefore characterization of SjD patients with ‘radiologic LIP’ might be even more clinically relevant. Moreover, radiologic and PFT findings of LIP patients in this study were comparable to those of previous reports of SjD-LIP with histologic confirmation [16, 29], further supporting their proper classification.

Another major strength of this study is the longitudinal follow-up, with a median duration of five years. This allowed us to assess the clinical course of SjD-LIP patients over time, suggesting a dissociation between radiologic progression and functional decline, and providing preliminary insight into long-term outcomes, including lymphoma.

From a clinical perspective, our findings support a phenotype-driven approach to the diagnosis and management of SjD-associated ILD. When LIP is suspected based on the respiratory manifestations, patients’ demographics, serologic features and concomitant extrapulmonary involvement, HRCT remains essential for diagnosis, particularly in light of the frequently preserved pulmonary function observed in this subgroup. Our longitudinal data further suggest that treatment decisions in SjD-associated LIP should primarily rely on respiratory symptoms, pulmonary function and systemic disease activity rather than on radiologic progression alone, as isolated cystic progression on HRCT did not translate into functional decline over time.

In conclusion, our findings suggest that LIP represents a distinct clinical and immunological phenotype within the spectrum of SjD-associated ILD. This subgroup is characterized by younger age at SjD onset, a higher prevalence of systemic and serologic markers of B cell hyperactivity, and a relapsing-remitting clinical course with good response to steroids and immunosuppressive treatment. Despite a favourable mid-term pulmonary outcome, with potential radiologic progression but preserved respiratory function, the constellation of B cell activation markers warrants long-term surveillance for lymphoproliferative complications and supports the rationale for considering B cell-targeted therapies in selected patients with systemic activity or recurrent inflammatory flares.

Future prospective studies are needed to clarify the effective risk of lymphoma development in LIP patients and the potential therapeutic role of B cell modulating agents.

## Supplementary Material

keag321_Supplementary_Data

## Data Availability

The data underlying this article will be shared on reasonable request to the corresponding author.
