# Supplementary material for: Lymphoid interstitial pneumonia in Sjögren disease: clinical course and comparison with other ILD patterns
Source: Rheumatology (Oxford). 2026 Jun 22;65(7):keag321. doi: 10.1093/rheumatology/keag321 (PMC13348713; doi:10.1093/rheumatology/keag321)
Supplement: keag321_Supplementary_Data [file keag321_supplementary_data.zip › 03-Jul-2026_015204_rhe-25-1885-File005.docx]

Supplementary material

**Supplementary Table S1**

| **Clinical features** | **LIP (n=11)** | **Non-LIP ILD (n=44)** | **p-value** |
| --- | --- | --- | --- |
| Xerostomia VAS (0-10), m (±SD) | 5.6 (±2) | 6.4 (±3.2) | 0.239 |
| Xeropthalmia VAS (0-10), m (±SD) | 6 (±2) | 5.6 (±3) | 0.652 |
| PG US Omeract score ≥ 2, n (%) | 7/9 (78) | 12/27 (44.4) | 0.443 |
| SM US Omeract score ≥ 2, n (%) | 7/9 (78) | 13/27 (48.1) | 0.251 |
| Fatigue VAS (0-10), m (±SD) | 6.6 (±3.4) | 6.7 (±2.2) | 0.442 |
| Pain VAS (0-10), m (±SD) | 4.7 (±3.5) | 5.7 (±3) | 0.187 |
| ESSPRI, m (±SD) | 5.7 (±2.4) | 6.2 (±2.2) | 0.867 |

**Supplementary Table S2**

| **Treatment** | **LIP (n=11)** | **Non-LIP ILD (n=44)** | **p-value** |
| --- | --- | --- | --- |
| Systemic glucocorticoids, n (%) | 10/11 (91) | 38/44 (86) | 1.00 |
| Any immunosuppressive therapy, n (%) | 7/11 (64) | 29/44 (66) | 0.88 |
| Cyclophosphamide, n (%) | 0/11 (0) | 11/44 (25) | — |
| Mycophenolate mofetil, n (%) | 1/11 (9) | 12/44 (27) | — |
| Azathioprine, n (%) | 1/11 (9) | 3/44 (7) | — |
| Rituximab, n (%) | 4/11 (36) | 3/44 (7) | — |
| Belimumab, n (%) | 1/11 (9) | 0/44 (0) | — |

Individual agents are reported descriptively due to small numbers, non-mutually exclusive regimens and sequential treatment strategies; therefore, p-values are not shown for single drugs.

**Supplementary Table S3** Summary of the SjD-related clinical and laboratory features of LIP patients

| **Pt** | **Sex** | **Age at SjD (LIP) diagnosis** | **Anti-Ro/La**  **autoAb** | **Sicca /fatigue/pain VAS (0-10)** | **PG/SG US Omeract score (0-3)** | **Extra-pulmonary involvement** | **Serologic markers** |
| --- | --- | --- | --- | --- | --- | --- | --- |
| 1 | F | 20 (69) years | Ro60/52/La+ | 8/4/7 | 3/3 | Constitutional, glandular (SGE), cutaneous (purpura), lymphnodal, haematological (lymphopenia) | RF+, cryo+, hyper-γ, low C3 |
| 2 | F | 27 (37) years | Ro60/52+ | 6/5/8 | 1/2 | Haematological (lymphopenia) | Hyper-γ |
| 3 | F | 51 (67) years | Ro60/52/La+ | 5/2/2 | 1/2 | Articular (infl. arthralgia) | RF+, hyper-γ, IgG k MC, low C3 and C4 |
| 4 | F | 37 (41) years | Ro60/52/La+ | 6/10/9 | 3/3 | Constitutional, Glandular (SGE), Renal (TIN) | RF+, hyper-γ, low C3 and C4 |
| 5 | F | 28 (65) years | Ro60/52/La+ | NA | NA | Glandular (SGE), cutaneous (purpura), lymphnodal, haematological (lymphopenia) | RF+, hyper-γ, IgG λ MC |
| 6 | F | 70 (69) years | Ro60/52/La+ | 3/0/0 | 3/3 | None | RF+, hyper-γ |
| 7 | F | 48 (58) years | Ro60/52/La+ | 7/9/6 | 0/0 | Cutaneous (purpura) | RF+, hyper-γ |
| 8 | F | 14 (50) years | Ro60/52/La+ | 8/8/1 | 2/3 | Constitutional, haematological (lymphopenia) | Hyper-γ |
| 9 | F | 32 (35) years | Ro60/52+ | 7/8/7 | 2/1 | Articular (infl. arthralgia), lymphnodal | RF+, hyper-γ, IgG k MC |
| 10 | F | 37 (54) years | Ro60/52/La+ | 5/9/7 | 3/3 | Glandular (SGE), lymphnodal, haematological (neutropenia, lymphopenia) | Hyper-γ, low C3 and C4 |
| 11 | F | 63 (64) years | Ro60/52+ | 2/8/0 | NA | Lymphnodal, haematological (neutropenia, lymphopenia) | Hyper-γ, low C3 and C4 |

AutoAb= autoantibodies; VAS= Visual Analogue Scale; PG= Parotid Glands; SG= Submandibular Glands; US= Ultrasound; SGE= Salivary Glands Enlargement; Infl. = Inflammatory; RF= rheumatoid factor; Hyper γ= hypergammaglobulinemia; MC= monoclonal component; TIN= tubule-interstitial nephritis

Presence of SGE and extra-glandular SjD manifestations is defined according to ESSDAI definitions.

**Supplementary Appendix**

FEF25–75% values were available in a subset of patients (7/11 LIP and 12/44 non-LIP). Individual values are reported below for completeness:
LIP: 89, 61, 90, 75, 84, 65, 84 (% predicted).
Non-LIP: 74, 94, 180, 141, 49, 70, 53, 92, 99, 97, 67, 70, 92 (% predicted).
Given the high proportion of missing data, no comparative analyses were performed.

**Supplementary Figures**

Representative HRCT images from each patient included in the study are presented below (one image per patient). Arrows highlight the main radiologic findings, described in a clockwise direction starting from the 12 o’clock position.


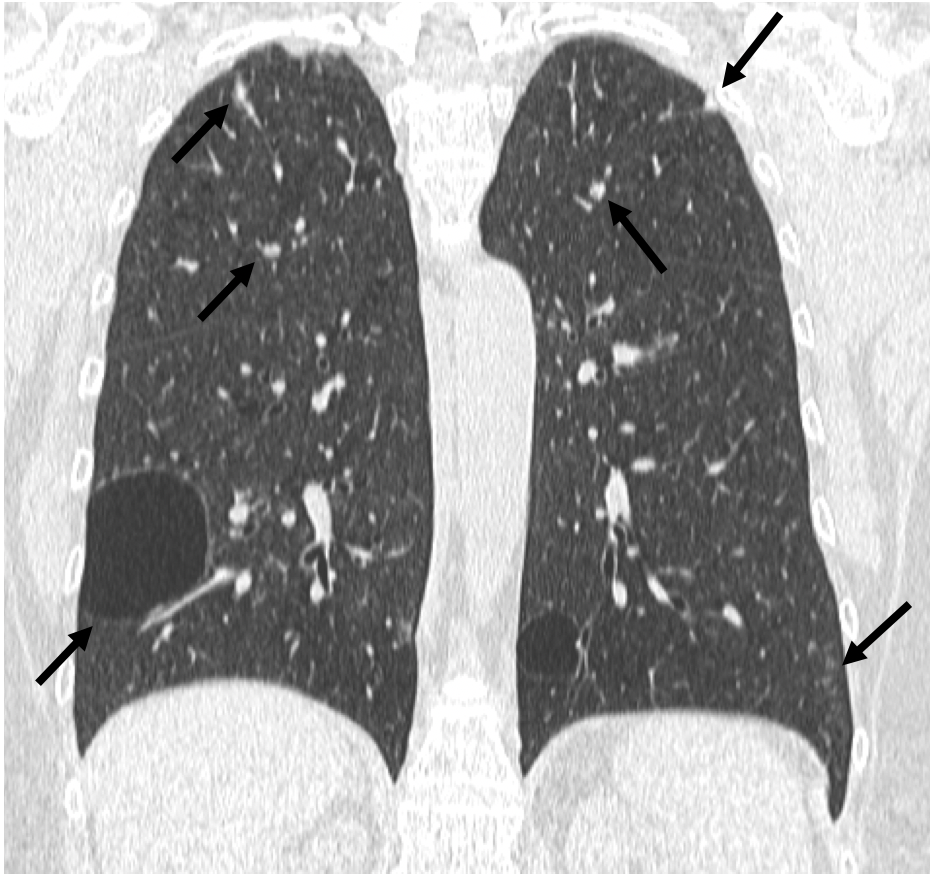


**Patient 1**. Arrows show subpleural and peribronchovascular micronodules (with a perilymphatic distribution pattern), inflammatory involvement of the small airways, and air cysts


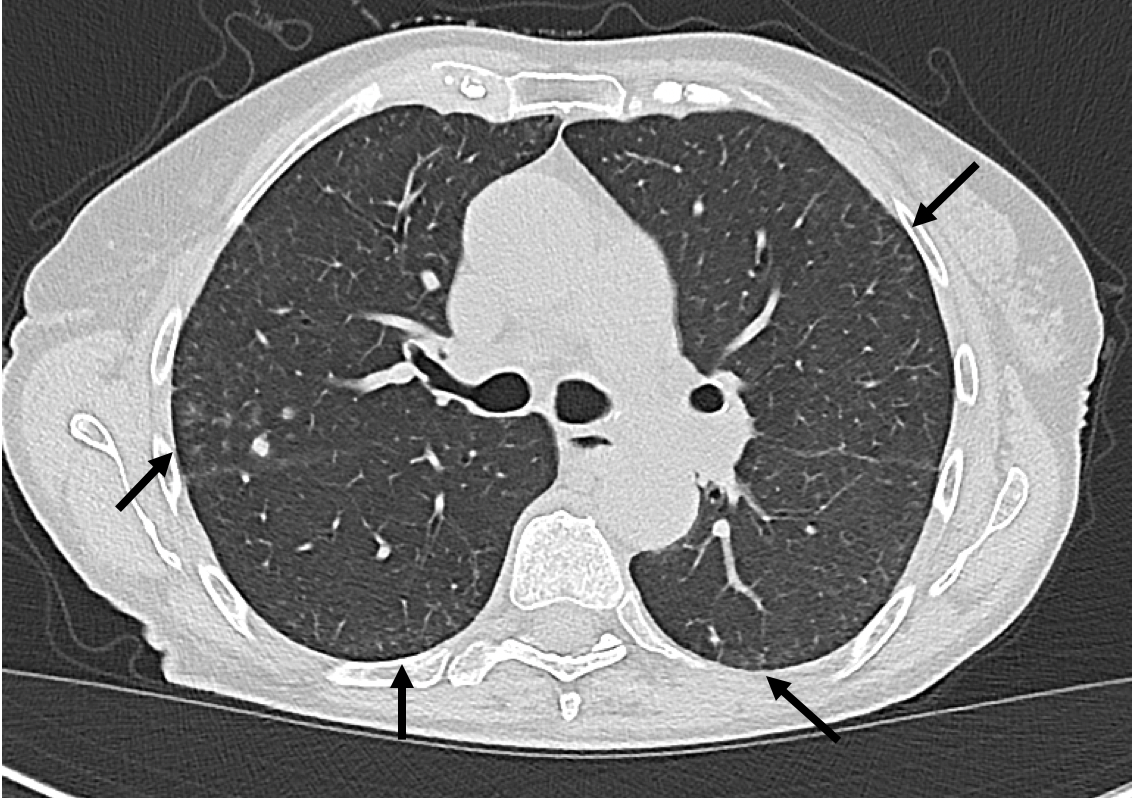


**Patient 2.** Arrows show inter- and intralobular septal thickening, subtle areas of increased parenchymal attenuation, and signs of small airway involvement in the dorsal segment of the right upper lobe.


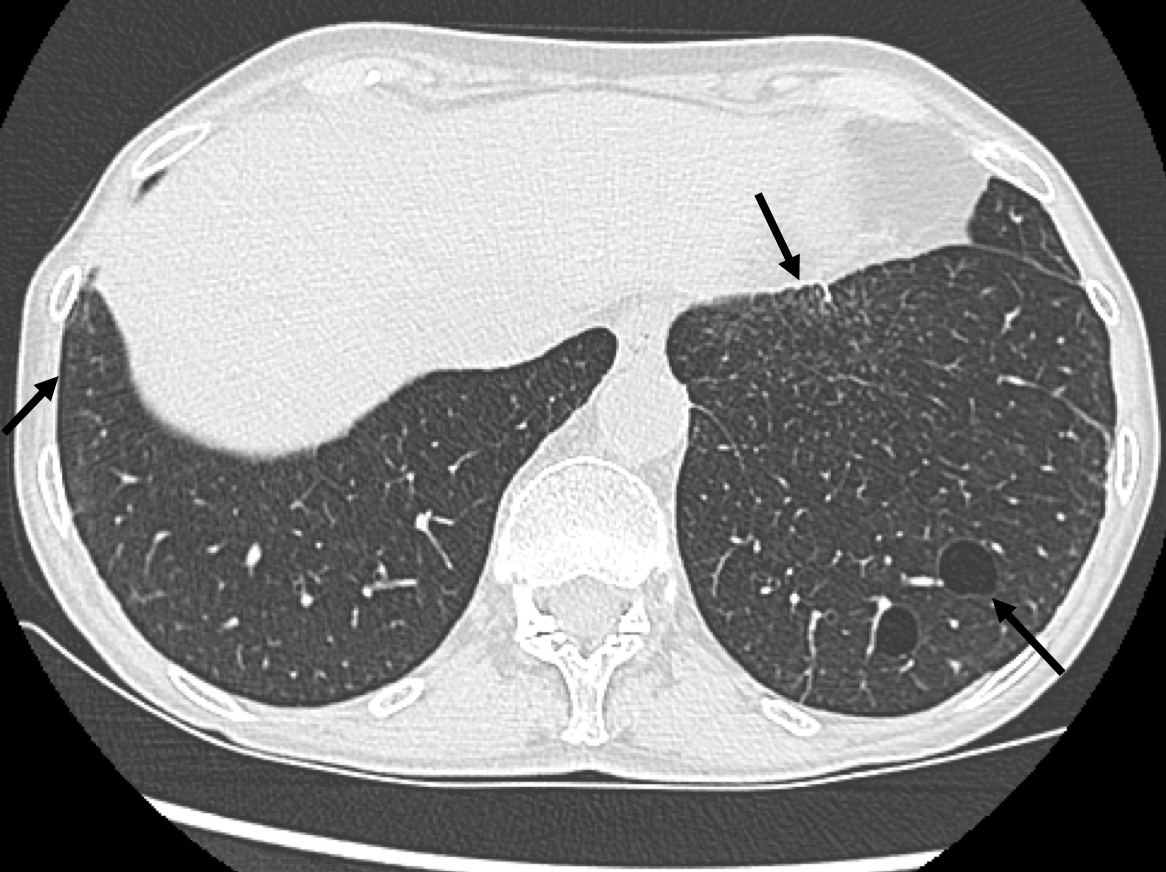


**Patient 3.** Arrows show the presence of small parenchymal air cysts and mild bronchiolar wall thickening, consistent with inflammatory involvement of the small airways.


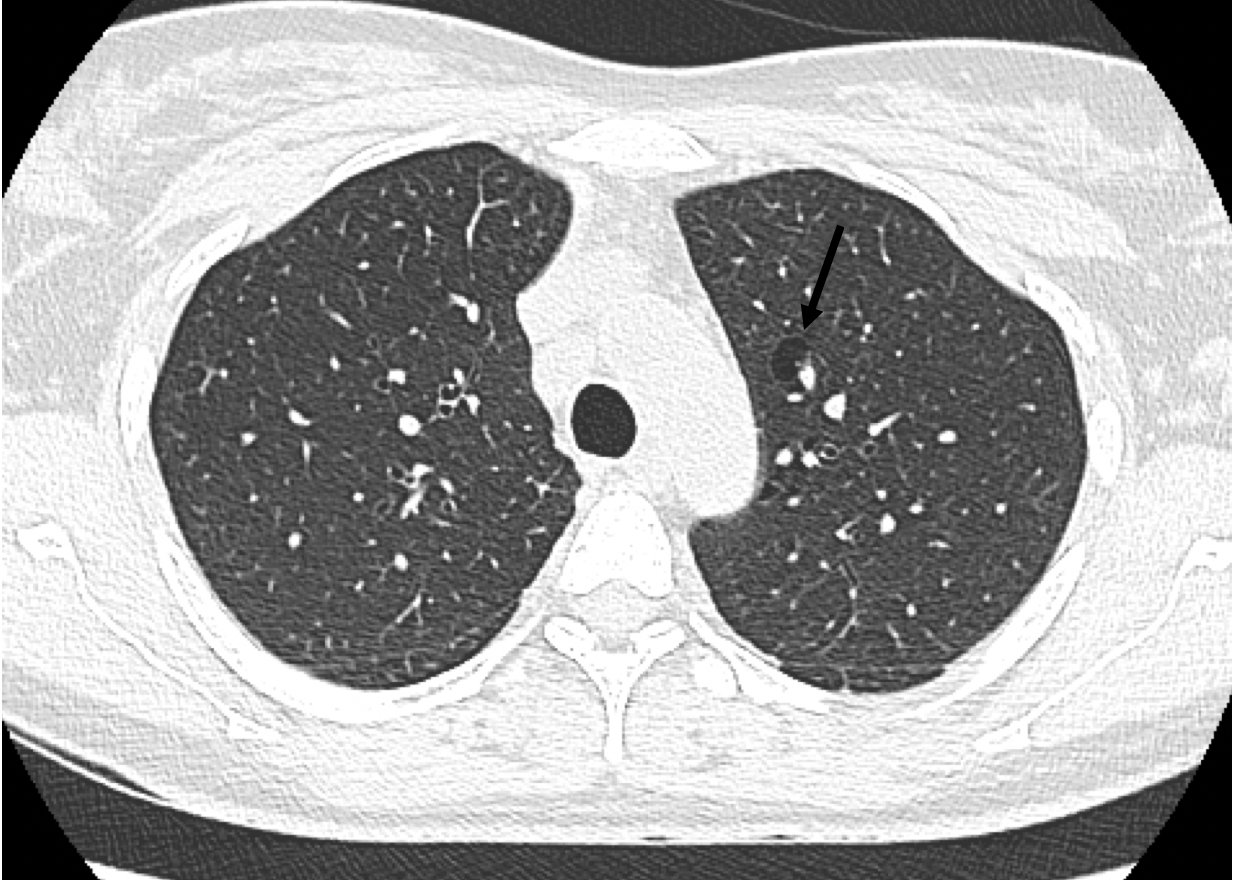


**Patient 4.** minimal parenchymal involvement, characterized by sporadic cysts (arrow) and mild, diffuse linear thickening of the bronchiolar walls.


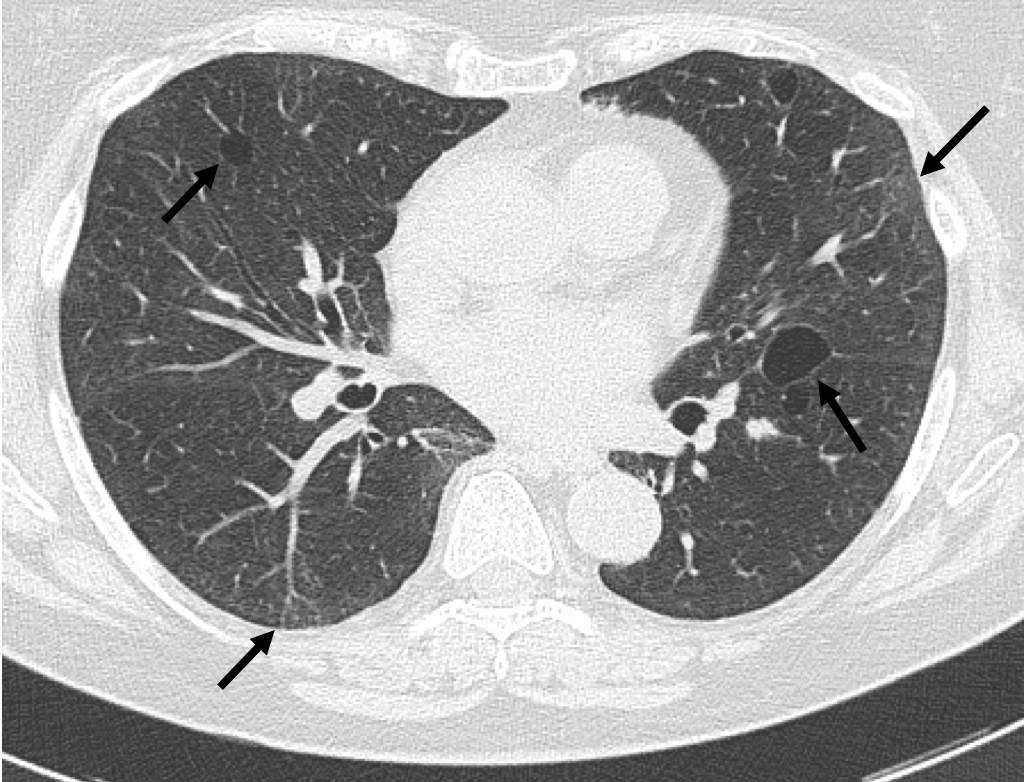


**Patient 5.** Arrows show the presence of ground-glass opacities associated with fine intralobular septal thickening, as well as a few thin-walled parenchymal air cysts.


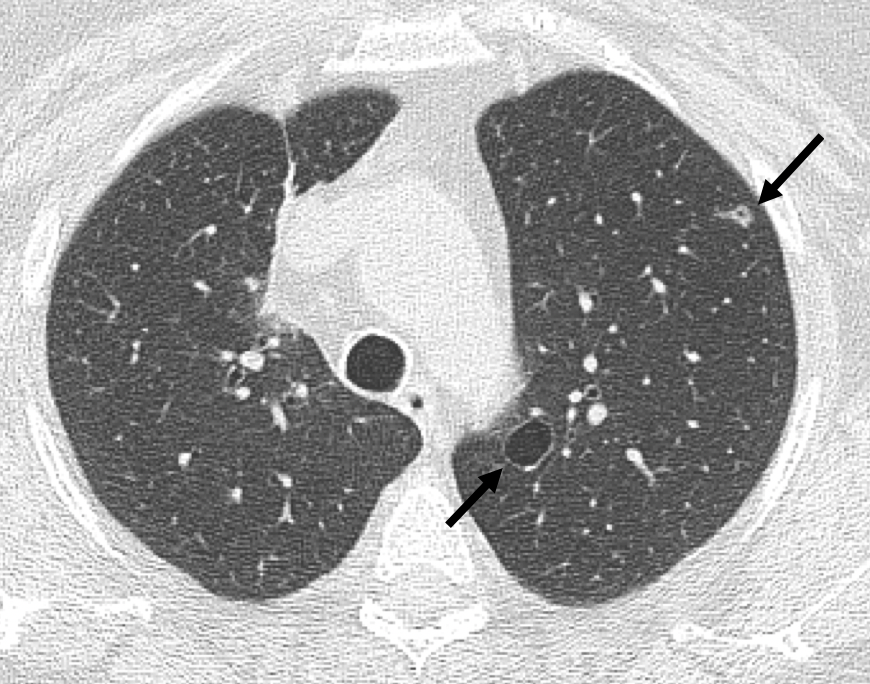


**Patient 6**. sporadic signs of small airway involvement and parenchymal cysts.


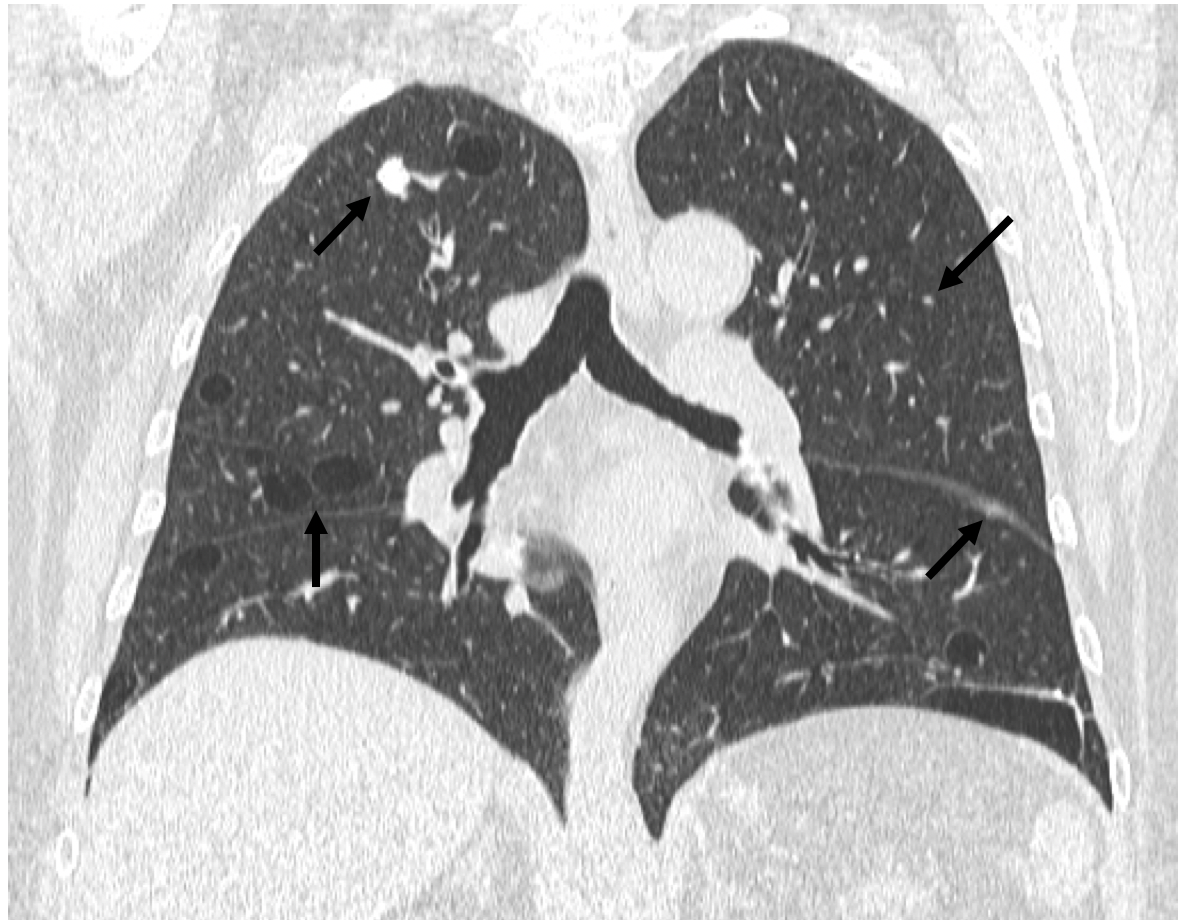


**Patient 7.** Arrows highlight the presence of micronodules and nodules with a perilymphatic and perifissural distribution, along with multiple bilateral parenchymal air cysts.


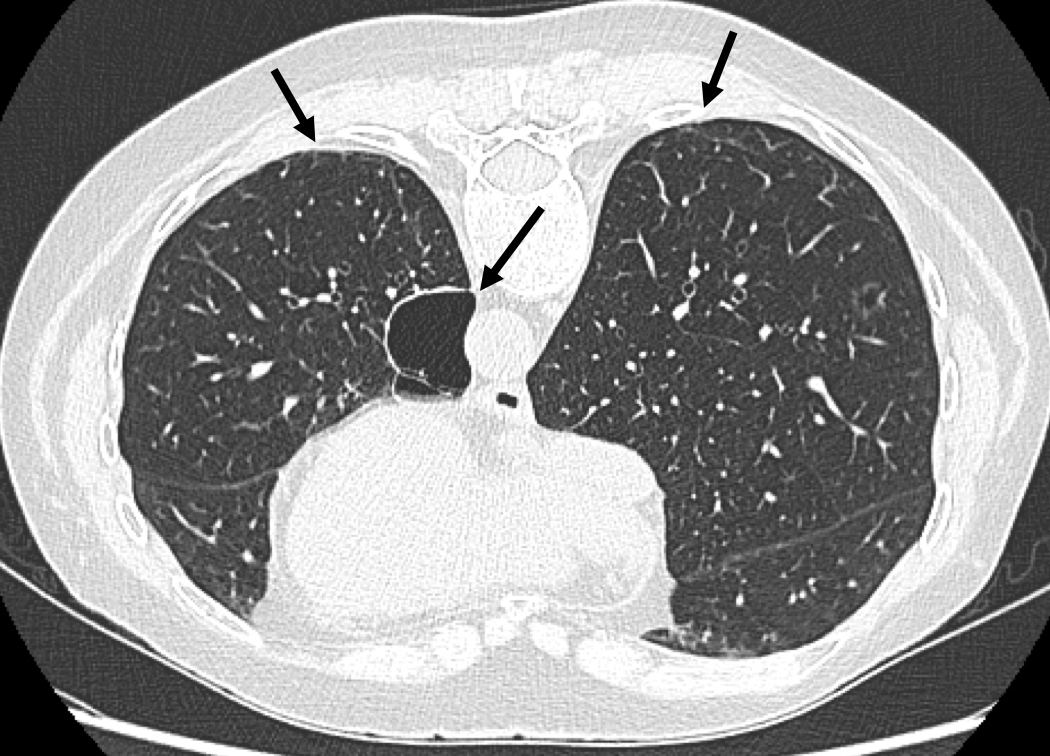


**Patient 8.** Arrows show fine septal thickening, predominantly interlobular, involving the dorsal regions of the lower lobes, as well as a large bilobed air cyst in the left lower lobe.


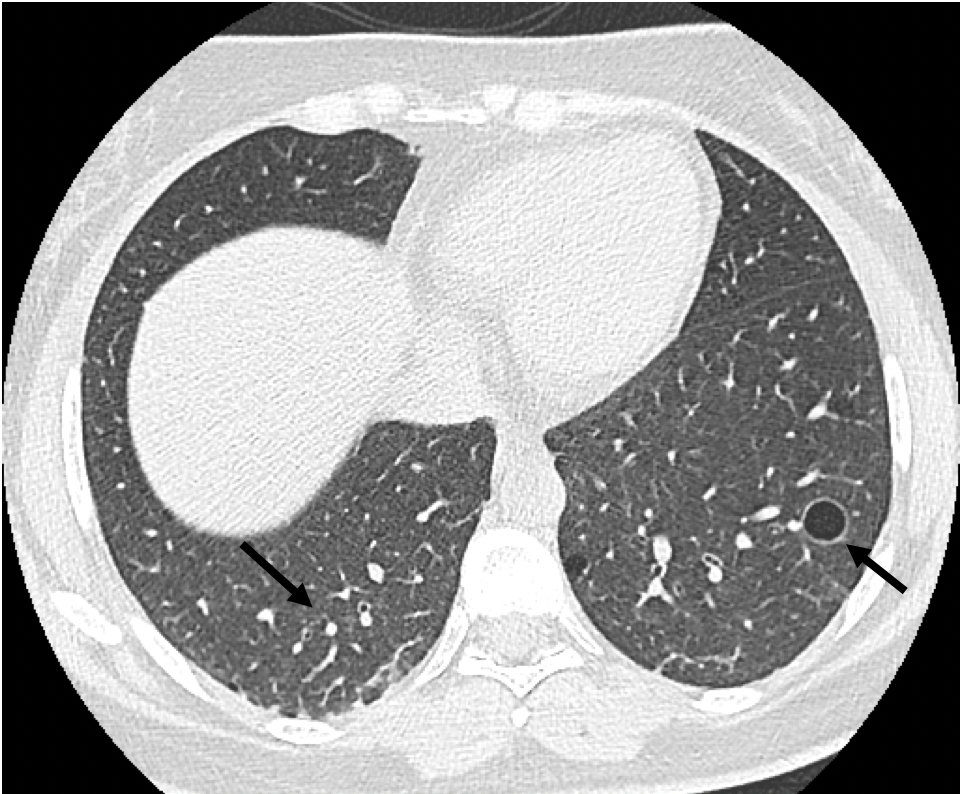


**Patient 9.** Arrows show the presence of small parenchymal air cysts and mild bronchiolar wall thickening, consistent with inflammatory involvement of the small airways.


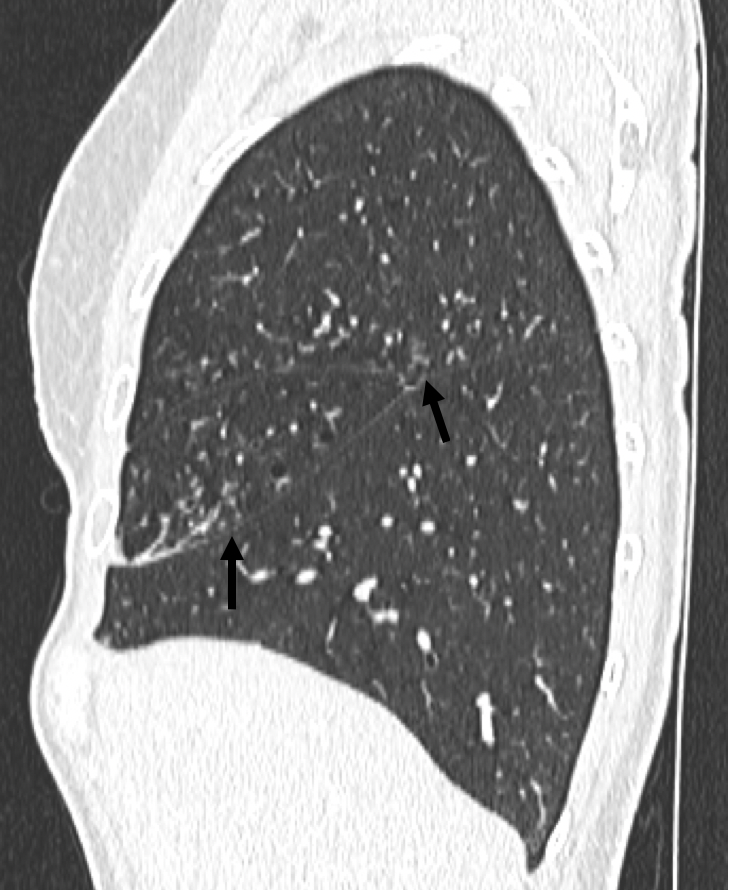


**Patient 10.** Arrows show the presence of branching micronodules and bronchiolar wall thickening in the right upper lobe and middle lobe, consistent with inflammatory involvement of the small airways.


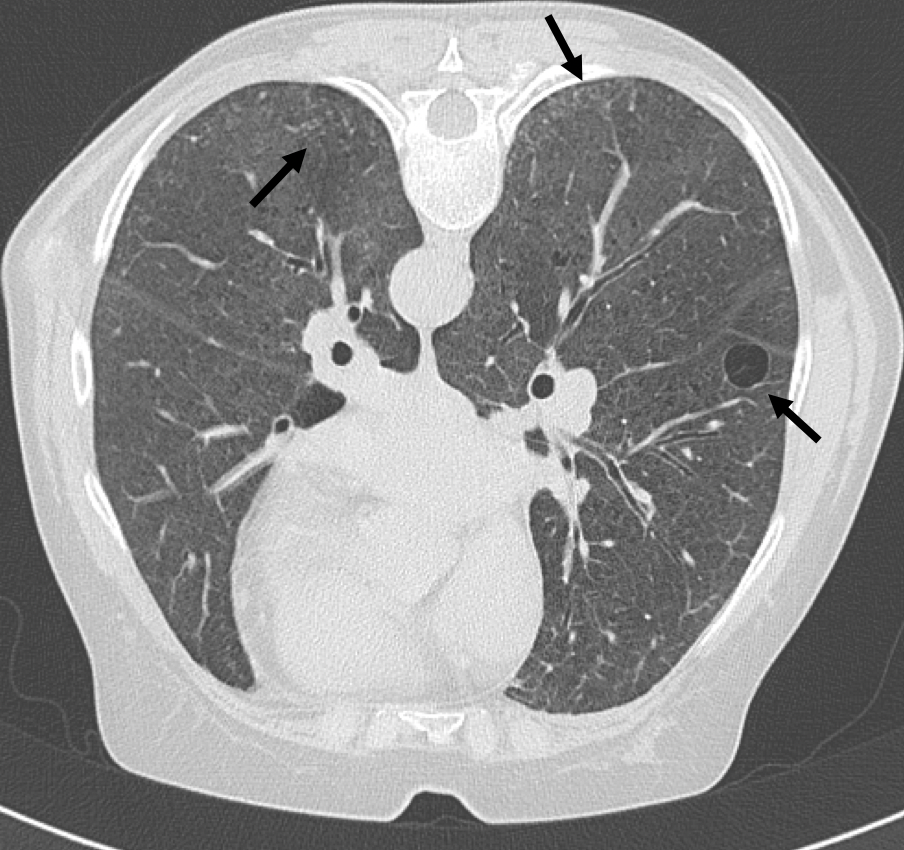


**Patient 11.** Prone acquisition showing diffuse areas of increased parenchymal attenuation; arrows indicate intralobular septal thickening, air cysts, and small airway involvement.
